# Peripheral facial nerve schwannoma at the inferior mandibular margin: a case report

**DOI:** 10.1093/jscr/rjab299

**Published:** 2021-07-31

**Authors:** Akiko Sakakibara, Takumi Hasegawa, Daisuke Takeda, Junya Kusumoto, Shunsuke Sakakibara, Masaya Akashi

**Affiliations:** Department of Oral and Maxillofacial Surgery, Kobe University Graduate School of Medicine, Kobe, Japan; Department of Oral and Maxillofacial Surgery, Kobe University Graduate School of Medicine, Kobe, Japan; Department of Oral and Maxillofacial Surgery, Kobe University Graduate School of Medicine, Kobe, Japan; Department of Oral and Maxillofacial Surgery, Kobe University Graduate School of Medicine, Kobe, Japan; Department of Plastic Surgery, Kobe University Graduate School of Medicine, Kobe, Japan; Department of Oral and Maxillofacial Surgery, Kobe University Graduate School of Medicine, Kobe, Japan

**Keywords:** peripheral facial nerve, schwannoma, mental foramen, marginal mandibular branch, percutaneous enucleation

## Abstract

Schwannomas commonly occur in the head and neck region as acoustic neuromas. Facial nerve schwannomas are rare and usually occur in the temporal region. A 57-year-old woman presented with a mass at the right mandibular margin. Magnetic resonance imaging revealed a schwannoma located immediately caudal to the mental foramen. We were initially uncertain whether it arose from the trigeminal nerve or the facial nerve. Excision was performed under general anesthesia. The mass was encapsulated and easily detached from the surrounding tissue. The nerve of origin was identified proximal to the tumor. A facial nerve origin was confirmed as the muscles supplied by the marginal mandibular branch of the facial nerve moved on nerve stimulation. Nerve fibers were not found distal to the tumor, possibly because they had been cut during excision. We believe that this is the first report of a schwannoma arising from the peripheral facial nerve.

## INTRODUCTION

Schwannomas are derived from Schwann cells, which are of ectodermal origin. They can arise from any part of a myelinated nerve. Nearly half of them occur in the head or neck [1, 2], although acoustic schwannomas, which arise from the eighth cranial nerve, also commonly occur. Extracranial schwannomas arising from the 7th, 8th and 12th cranial nerves are commonly observed in the parapharyngeal space and neck [3–5].

Peripheral facial nerve schwannomas are rare. Here, we report the case of a 57-year-old woman with an enucleated submandibular schwannoma derived from the peripheral facial nerve, and then, we discuss the relevant literature. We believe this is the first report of a schwannoma originating from the peripheral facial nerve at the mandibular margin.

## CASE REPORT

A 57-year-old woman visited our hospital in December 2019 for swelling and induration at the right mandibular margin. She had undergone surgery for bilateral metachronous breast cancer between 10 and 17 years ago. She first noticed the mass 1 year previously and had been following up at a nearby hospital. She was eventually referred to our hospital due to an increase in mass size.

**Figure 1:**
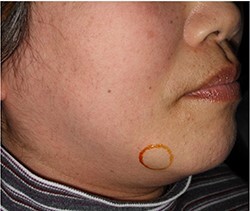
Extraoral findings; a 15-mm indurated mass is seen at the right mandibular margin.

**Figure 2:**
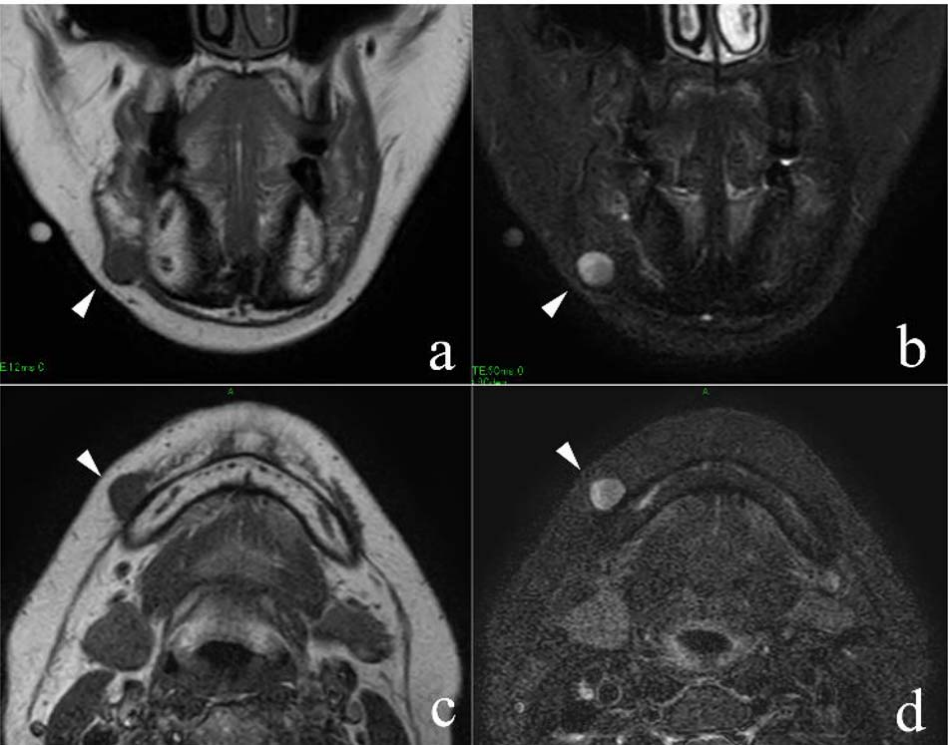
MRI findings; the arrowheads indicate the schwannoma; (**a**) low signal intensity on T1-weighted coronal image; (**b**) high signal intensity on T2-weighted coronal image; (**c**) low signal intensity on T1-weighted axial image; (**d**) high signal intensity on T2-weighted axial image.

**Figure 3:**
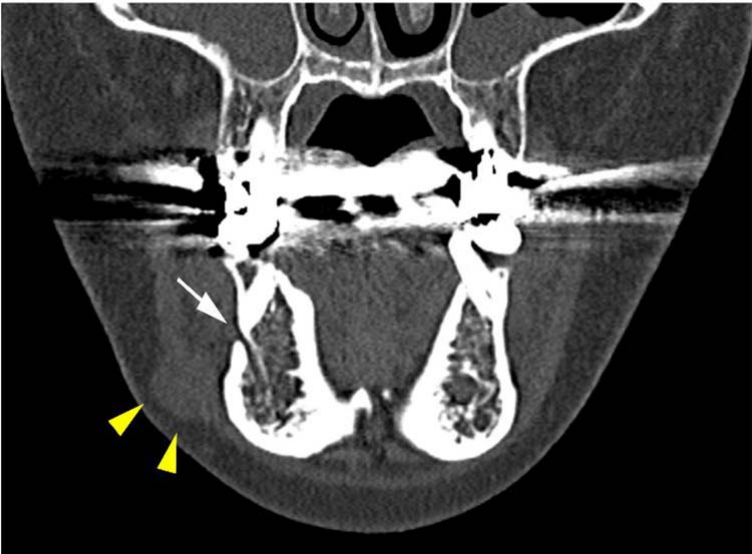
CT findings of a coronal section; yellow arrowheads indicate the schwannoma; the white arrow indicates the mental foramen.

She had a 15-mm, painless, non-pitting and indurated mass at the right mandibular margin (Fig. 1). Oral examination revealed no mucosal abnormalities or swelling. The mass was difficult to palpate from the oral side due to the presence of buccal frenulum on the mucosa over the mass. Magnetic resonance imaging (MRI) revealed a 15-mm nodule with a clear border at the right mandibular margin, with low and high signal intensities on T1- and T2-weighted images, respectively (Fig. 2). Computed tomography (CT) revealed a 15-mm low-density nodule in the right mandibular margin. Calcification, bone invasion or lymph node enlargement were not observed (Fig. 3). The mass was radiologically diagnosed as an epidermoid cyst. The differential diagnoses were schwannoma, neurofibromatosis, pleomorphic adenoma and a powdery mass; however, schwannoma was considered to be most likely.

**Figure 4:**
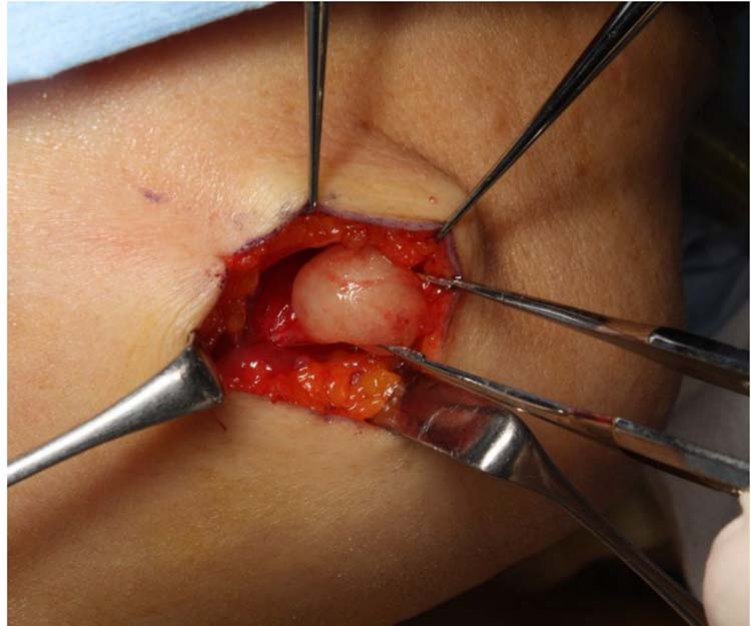
Intraoperative findings of tumor enucleation; the marginal mandibular branch of the facial nerve, which was thought to be the nerve of origin, was seen proximal to the tumor.

**Figure 5:**
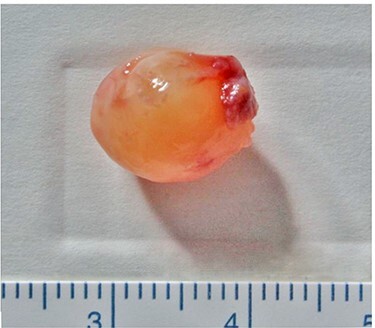
Excised mass; it is a yellowish-white, spherical, encapsulated, solid mass.

The mass was excised under general anesthesia. A 25-mm incision was made at the right mandibular margin, and the upper part of the tumor was dissected. The tumor was covered by the thin platysma muscle. Neurostimulation was performed to ensure that the marginal mandibular nerve was preserved while entering the platysma muscle, and the tumor periphery was detached. The tumor was encapsulated, smooth, elastic and hard. Right chin and lip movements were observed on nerve stimulation after tumor detachment, with no movement when the tissue under the mass was stimulated. A nerve arising from the marginal mandibular branch of the facial nerve was identified proximal to the tumor (Fig. 4). The nerves distal to the tumor could not be identified; these funicular nerves might have been cut during detachment. A 13- × 10-mm, spherical, yellowish-white, solid mass was excised following central nerve ligation (Fig. 5).

**Figure 6:**
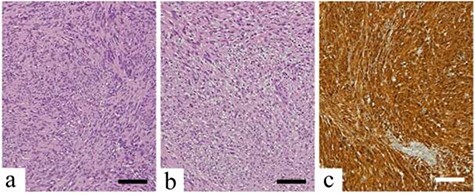
Histopathological findings; scale bars indicate 100 μm (**a**) Antoni Type A tissue with regular arrangement of tumor cells with spindle-shaped and elongated nuclei; (**b**) Antoni Type B tissue with spindle-shaped cells in an edematous and myxomatous background; (**c**) the neoplastic cells are strongly stained with S-100 protein.

Gross examination revealed a 12- × 10- × 10-mm^3^, solid, encapsulated tumor. Histopathological examination revealed tumor cells with elongated spindle-shaped nuclei and a regular, palisading Antoni A pattern (Fig. 6a). Antoni B tissue (Fig. 6b) and loose spindle cells were observed in the edematous and myxomatous background. The tumor cells were positive for S-100 (Fig. 6c), as typically seen in benign schwannomas.

Post-operative edema temporarily hindered right lower lip movement, with improvement after 2 days. Facial movements were bilaterally symmetrical. Six months post-operatively, Semmes–Weinstein testing revealed normal responses to the 1.65 and 2.36 filaments at the areas supplied by the inferior labial, oral angular and mental branches of the mandibular nerve, with no left–right difference. One year post-operatively, the patient had no recurrence.

## DISCUSSION

Schwannomas can arise from any cranial nerve except the olfactory and optic nerves [6]. It has a high incidence (25–45%) in the head and neck [7] and rarely occurs in the maxillary region. It is usually slowly progressive, asymptomatic and benign.

Schwannomas are usually well-defined, round and smooth-edged masses. Macroscopically, they resemble fibromas, lipomas, epithelial hyperplasia, hemangiomas, granulocytomas, neurofibromas, neuromas, leiomyomas, rhabdomyomas, myxocysts and salivary gland tumors.

Hatziotis *et al*. found that most oral tumors developed in the tongue (55.7%), followed by the palate (10.4%), floor of the mouth (9.4%), buccal mucosa (8.50%), gingiva (5.7%) and lip (5.7%) [8]. Gallo *et al*. reported the tongue as the most common tumor site (45.2%), followed by the buccal mucosa (13.4%), mandible (11.5%), floor of mouth (8.9%), palate (7.6%), gingiva (4.5%) and lip (4.5%) [9].

Facial nerve schwannomas often arise intracranially and intratemporally [10, 11], extending into the facial nerve canal or near the stylomastoid foramen. Nerve compression by the tumor, bone and surrounding connective tissue results in facial nerve palsy. The incidence of facial nerve schwannoma in the parotid gland is 9–14% [10].

Previous reports of subcutaneous schwannomas of the face did not mention the nerve of origin. Tumors of the upper lip and ala nasalis and those located lateral to the angle of the mouth and around the mental foramen have been reported [12–15]. In two of these patients [13, 15], the mental nerve was preserved during tumor resection; sensory recovery occurred, possibly because the tumor was separated from the nerve. However, these tumors originated from the mandibular nerve and were in the oral cavity, not the mental foramen. There have been no reports of peripheral facial nerve schwannomas.

Patients with subcutaneous schwannomas often experience no neurologic symptoms, although intraoperative nerve damage may cause neurologic deficits. Therefore, the surgical approach should be carefully selected. In this case, the nerve of origin was a branch of either the mental nerve (sensory nerve) or the facial nerve (motor nerve). The mass was caudal to the mental foramen. If an oral approach is selected and an oral vestibular incision is made, a mental foramen may be encountered intraoperatively, leading to mental nerve palsy. However, a transcutaneous incision may damage the marginal mandibular nerve. After discussing with our patient, we adopted a percutaneous approach, and nerve damage was avoided.

Intraoperative nerve stimulation showed that the nerve of origin of our patient’s tumor was the facial nerve. Since it originated from the nerve periphery (terminal part of the marginal mandibular branch), there was no neurological deficit.

## CONFLICT OF INTEREST STATEMENT

None declared.

## FUNDING

This research did not receive any specific grant from funding agencies in the public, commercial, or non- profit sectors.
